# Single-graded CIGS with narrow bandgap for tandem solar cells

**DOI:** 10.1080/14686996.2018.1444317

**Published:** 2018-03-16

**Authors:** Thomas Feurer, Benjamin Bissig, Thomas P. Weiss, Romain Carron, Enrico Avancini, Johannes Löckinger, Stephan Buecheler, Ayodhya N. Tiwari

**Affiliations:** a Laboratory for Thin Films and Photovoltaics, Empa – Swiss Federal Laboratories for Materials Science and Technology, Duebendorf, Switzerland

**Keywords:** Photovoltaics, CIGS, narrow bandgap, CIS, thin-film solar cells, tandem solar cells, 50 Energy Materials, 209 Solar cell / Photovoltaics

## Abstract

Multi-junction solar cells show the highest photovoltaic energy conversion efficiencies, but the current technologies based on wafers and epitaxial growth of multiple layers are very costly. Therefore, there is a high interest in realizing multi-junction tandem devices based on cost-effective thin film technologies. While the efficiency of such devices has been limited so far because of the rather low efficiency of semitransparent wide bandgap top cells, the recent rise of wide bandgap perovskite solar cells has inspired the development of new thin film tandem solar devices. In order to realize monolithic, and therefore current-matched thin film tandem solar cells, a bottom cell with narrow bandgap (~1 eV) and high efficiency is necessary. In this work, we present Cu(In,Ga)Se_2_ with a bandgap of 1.00 eV and a maximum power conversion efficiency of 16.1%. This is achieved by implementing a gallium grading towards the back contact into a CuInSe_2_ base material. We show that this modification significantly improves the open circuit voltage but does not reduce the spectral response range of these devices. Therefore, efficient cells with narrow bandgap absorbers are obtained, yielding the high current density necessary for thin film multi-junction solar cells.

## Graphical abstract

**Figure:**
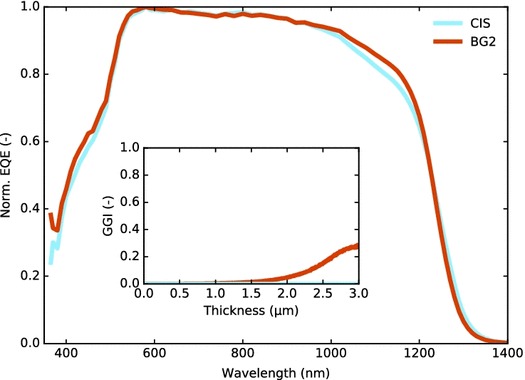

## 1. Introduction

With continuously declining prices of solar modules, the improvement of solar cell efficiency has become a very important subject to further reduce the cost of the full photovoltaic (PV) system. In recent years, Cu(In,Ga)Se_2_ (CIGS) research has led to impressive solar cell efficiencies up to 22.6% [1]. With single-junction solar cells getting closer to their maximal efficiency limit [2], the alternative concept of multi-junction (or tandem) solar cells is an interesting option for higher efficiencies surpassing the Shockley–Queisser limit [3–5]. Recent developments of high efficiency perovskite solar cells, especially semitransparent ones, open the opportunities for all thin film tandem solar modules on large and inexpensive substrates.

For tandem solar cells with two junctions, one can develop devices in 4-terminal configuration (both cells connected individually) or in 2-terminal monolithic configuration (both cells connected in series). The 4-terminal device offers the advantage of a higher tolerance in choice of sub-cell bandgaps to reach high efficiencies and of ease of production due to the individual nature of the sub-cells. The monolithic configuration has reduced parasitic absorption as well as the advantage of utilizing only a single substrate and electrical circuit. But due to the series connection the current of both cells has to match. Therefore, the suitable bandgap values for the cells are more constrained, with an absolute optimum at 0.94 eV for the bottom cell and 1.60 eV for the top cell [6]. CIGS thin film solar cells are highly promising as bottom cells due to remarkably high efficiency and the tunable nature of the absorber bandgap. While CIGS with state of the art composition works well for 4-terminal tandems, the current matching constraint for monolithic devices requires a reduction of bandgap towards CuInSe_2_ (CIS, ~1.0 eV).

While most of the development work has focused on the high efficiency CIGS with a bandgap suitable for single junction cells, narrow bandgap CIS has been lagging behind, keeping record efficiency at 15.0% [7].

A comparison of photovoltaic parameters of record efficiency devices (Table 1) highlights a large discrepancy between theoretically achievable and practically achieved values for CIS, especially in the open circuit voltage (*V*_OC_). By comparison, the CIGS record solar cell is considerably closer to the theoretical maximum. In such high performing CIGS cells, the absorber layers consists of a material with varying gallium to indium ratio across the thickness of the absorber, resulting in a complex bandgap grading profile. Most generally those profiles present increased Ga concentrations towards the front and the back of the absorber, the so-called double grading. Integrated relative Ga concentrations slightly above 0.3 ([Ga]/([Ga] + [In]), called GGI) are employed, corresponding to average bandgap of 1.15 eV, and the optical minimum bandgap of the absorber is located at around 1.1 eV [10,11]. The compositional gradings have been reported to reduce recombination at the back contact and have become an important feature of high efficiency CIGS cells [12–14].

**Table 1.** Comparison of bandgap (*E*_g_), open circuit voltage (*V*_OC_), short circuit current (*J*_SC_), fill factor (FF) and efficiency (Eff.) for world record (WR) solar cells [1,7,8] with theoretically calculated values (SQ) [9], derived from detailed balance calculations for AM1.5G irradiation [2]. Bandgaps for the record solar cells are estimated based on the published EQE data. The values in brackets represent the percentage of the theoretical limits.

	*E*_g_	*V*_OC_ (mV)	*J*_SC_ (mA cm^−2^)	FF (%)	Eff. (%)
CIS WR	~1.00	491 (66%)	40.6 (84%)	75.2 (88%)	15.0 (49%)
	*V*_oc_-loss	~510			
CIS SQ	1.00	749	48.2	85.4	30.8
CIGS WR	~1.14	741 (84%)	37.8 (88%)	80.6 (93%)	22.6 (69%)
	*V*_oc_-loss	~400			
CIGS SQ	1.14	879	42.9	87.1	32.8

In view of the application of CIS for tandem solar cells we aimed to improve the *V*_OC_ of CIS solar cells by implementing a single Ga back grading while keeping the spectral response of the devices extended to the 1.0 eV value. This paper presents our results with this approach and their relevance for perovskite/CIGS tandem solar cells.

## 2. Experimental section

### 2.1. Absorber deposition

Molybdenum coated soda lime glass was used as substrate. The CIGS absorber layer was grown by multistage co-evaporation of the constituent elements at a maximal substrate temperature around 500 °C. The compositional grading was introduced by changing the amount of Ga deposited in the first 20% of the growth. After deposition of the CIGS layer a post deposition treatment with NaF, followed by a 20 min Se annealing at a substrate temperature of about 350 °C was applied. All devices were completed with a CdS buffer layer by chemical bath deposition and nominally undoped and aluminum-doped zinc oxide front contacts deposited by RF magnetron sputtering. A Ni/Al grid was deposited by electron beam evaporation. The cell size of approximately 0.57 cm^2^ was defined by mechanical scribing. A description of the general process can be found in [15].

### 2.2. Electrical measurements

Current–voltage (IV) characteristics were measured under simulated AM1.5G illumination (class ABA solar simulator) at standard test conditions (1000 Wm^−2^, 25 °C) using a Keithley 2400 source meter in 4-terminal sensing. The illumination intensity was calibrated using a silicon solar cell from the Fraunhofer Institute for Solar Energy Systems. The spectral mismatch correction factor was 1.001 for all devices discussed here as calculated by comparison of the locally measured and the Fraunhofer-certified short circuit current (*J*_SC_) of a cell with equal bandgap to the cells here.

External quantum efficiency (EQE) was measured using a chopped light signal from a halogen light source, wavelength-selected with a double-grating monochromator. A halogen lamp bias with about 0.2 sun intensity was applied during the measurements. Certified Si and Ge cells were used for calibration. Since the measurement spot during EQE measurement cannot cover the whole cell, the grid shading was different between EQE and IV measurements. The EQE curves were scaled to match the respective *J*_SC_ in order to be representative.

Temperature dependent IV (T-IV) and *V*_OC_-*T* were measured in vacuum, using halogen illumination attenuated by neutral density (ND) filters for *J*_SC_-*V*_OC_ measurements. All measurements used 4-terminal sensing, and the measurement equipment was a Keithley 2400 source meter. The sample stage was temperature controlled using liquid nitrogen and heating elements.

### 2.3. Absorber characterization

The average composition of the different devices was determined by X-ray fluorescence (XRF) on an in-house built system using an Rh tube target at 45 keV and using a CIGS reference with known composition. This composition data were then used to scale the relative height in the time of flight secondary ion mass spectrometry (TOF-SIMS).

Thickness and morphology was determined using a Hitachi S-4800 scanning electron microscope (SEM).

To measure the optical properties of the absorber, as well as reflection of the full cells, a Shimadzu UV-3600 UV–vis spectrometer was used. The absorbers for these measurements were grown regularly on Mo/SLG and then detached by gluing them to a second sheet of glass using 3 M Scotch-Weld DP-100 epoxy and force the two sheets apart mechanically.

For time resolved photoluminescence (TRPL) measurements a 639 nm diode laser with pulse duration of ~100 ps was used as excitation source and an InGaAs photomultiplier in combination with time correlated single photon counting electronics was used for signal processing. Pulse repetition rate was approximately 1 MHz and the spot size in the 50 μm range as determined with a beam profiler. The typical photon density per pulse was around 7 × 10^11^ cm^−2^ as estimated from total laser power measurements. In all TRPL measurements the front of the absorber was passivated with a thin layer of CdS.

## 3. Results and discussion

### 3.1. Absorber films

**Figure 1.**
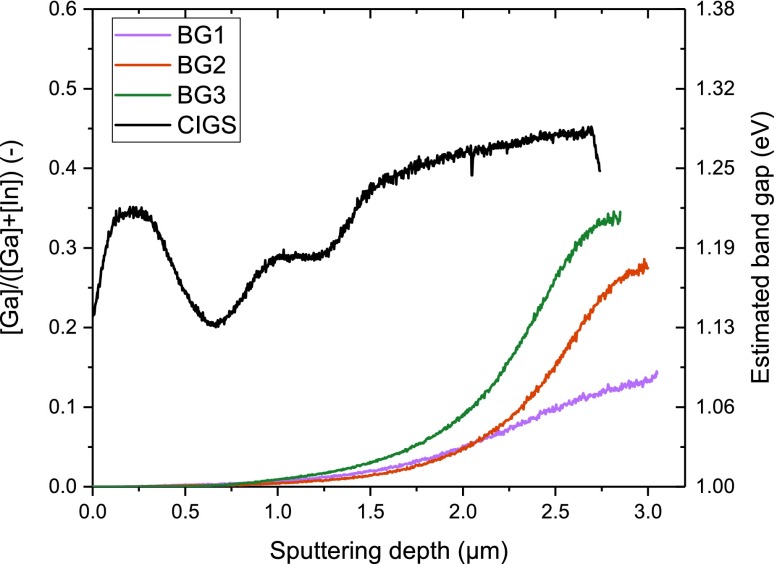
TOF-SIMS measured [Ga]/([Ga] + [In]) profile and the corresponding bandgap through the absorber. For comparison the GGI profile for a high efficiency CIGS sample is also included, while more details on this sample can be found in [15].

CIGS solar cells with a wide front region of Ga free CIS and different bandgap gradings towards the Mo back contact were prepared by a modified three stage co-evaporation process. The different compositional gradings produced here are named BG1, BG2 and BG3 in the order of increasing Ga content. The depth dependent composition profiles of such layers are shown in Figure 1. The total Ga content in all cells is below 10 at.% as determined by XRF.

All BG samples have a lower Ga content towards the back than a regular multi stage CIGS cell [16]. The bandgap increase between the point of lowest corresponding bandgap and the back interface is smaller (BG1), equal (BG2) or larger (BG3) compared to the reference CIGS.

**Figure 2.**
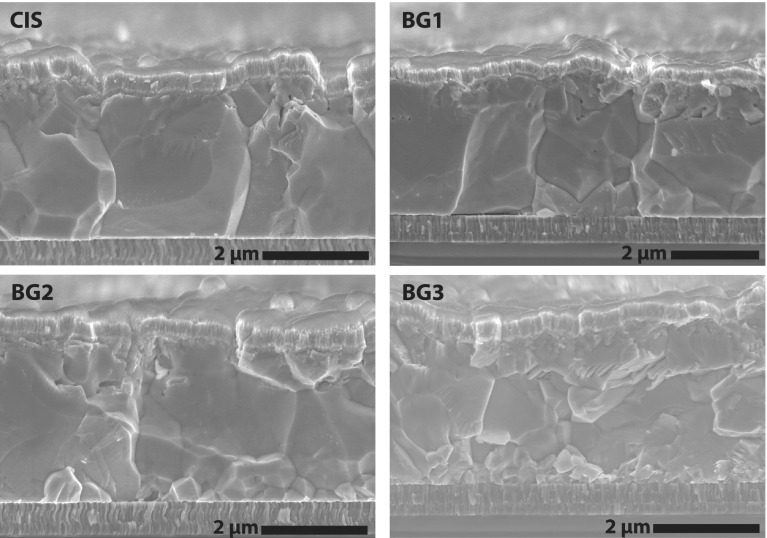
SEM micrographs of solar cell devices with different Ga back gradings. Increasing Ga content (CIS to BG3) leads to reduced grain size towards the back of the absorber.

The microstructure of the absorbers, as observed in SEM micrographs shown in Figure 2, changes from large-grained for pure CIS to considerably smaller grains in the case of the strongly graded absorbers. The thickness of the different absorber layers has been extracted from the SEM micrographs as 2.9 μm (CIS), 3.1 μm (BG1), 3.0 μm (BG2) and 2.9 μm (BG3) with a roughness of around 0.3 μm over multiple grains for all absorbers.

For all compositions there are distortions visible in the area where the third stage of absorber layer deposition starts. In the case of the graded cells a region of smaller CIGS grains is visible near the back contact, which is in line with previous reports describing a decrease in grain size with increasing Ga content [17].

### 3.2. Cell performance

**Figure 3.**
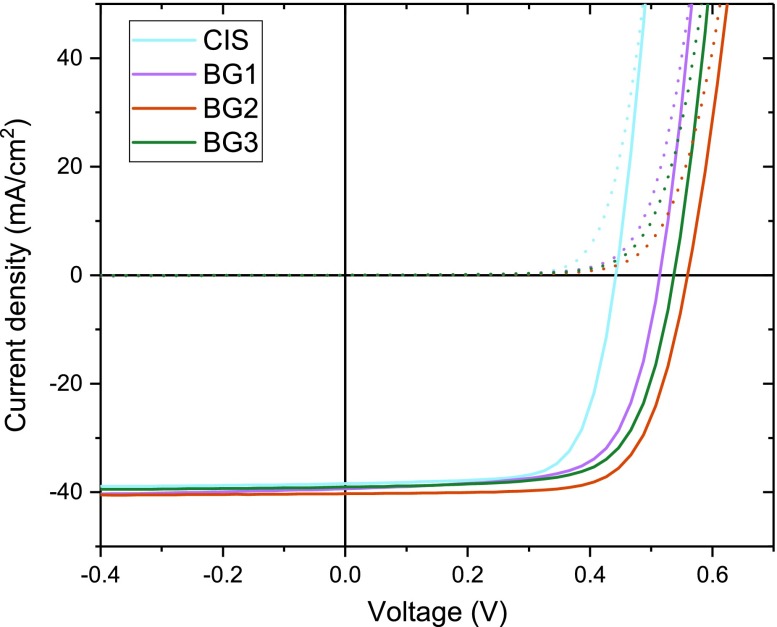
Dark (dotted) and illuminated (solid) IV-curves of CI(G)S solar cells.

The addition of a back surface grading improves the open circuit voltage of the cells considerably (Figure 3 and Table 2) compared to the Ga-free CIS reference.

**Table 2.** Photovoltaic parameters of the highest efficiency cell for each back surface graded sample.

	*V*_OC_ (mV)	*J*_SC_ (mA cm^−2^)	FF (%)	Eff. (%)
CIS	444	38.4	70.4	12.0
BG1	519	39.6	68.2	14.0
BG2	558	40.4	71.2	16.1
BG3	537	39.1	69.0	14.5

In addition, a notable increase in photocurrent (*J*_SC_) is observed for all cells with Ga grading resulting in a high value of 40.4 mA cm^−2^ (designated illumination area) and a photovoltaic conversion efficiency of 16.1% for BG2. The fill factor is somewhat lower for all cells with no clear trend to link to bandgap grading profile. We fitted the IV curves using a one diode model. The cells show pronounced differences in diode quality factor and low shunt resistance (Rp) under illumination which can lead to such differences in fill factor (SI T1). The general lower performance of BG3 may be related to a reduced doping and the small-grained morphology. Based on the results below no additional benefit is expected for this composition and hence no additional investigation was conducted. A statistical overview of all cells on the substrate for each back surface grading can be found in the supplementary information (SI F1), showing the same trends as given in Table 1 and Figure 1.

In the following, we discuss the observed gains in *J*_SC_ and *V*_OC_ in more detail and assess the suitability of the cells for tandem applications.

**Figure 4.**
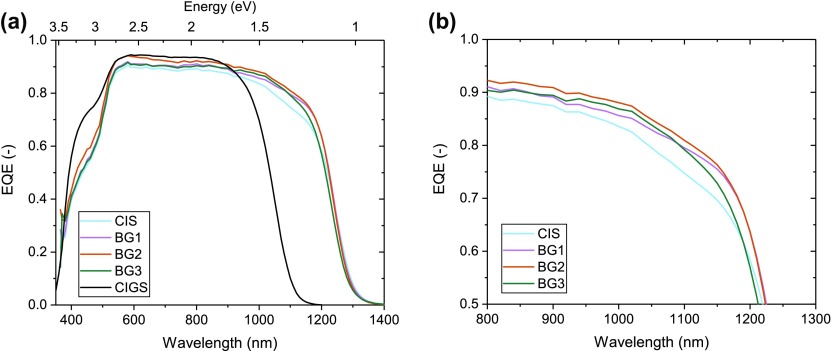
(a) External quantum efficiency of the back-graded devices. The black curve shows a typical CIGS reference; (b) close-up of the near infrared response.

The EQE curves of different cells are shown in Figure 4(a). The close-up (Figure 4(b)) shows that the increase in current for the back graded cells is the result of an improved EQE response in the near infrared (NIR) region. This can be explained by an improved collection efficiency for photogenerated carriers deeper in the absorber compared to the pure CIS. The addition of Ga leads mainly to an increase of the conduction band [18] and therefore generates an additional field for photogenerated electrons. This field induces a drift within the quasi neutral region towards the space charge region, where the electrons can be extracted and contribute to the *J*_SC_ [19]. The cells have a different ratio between cell area and grid shading, resulting in offsets of the respective EQE curves. The difference lies below 2% and does not alter the conclusions in this work.

**Figure 5.**
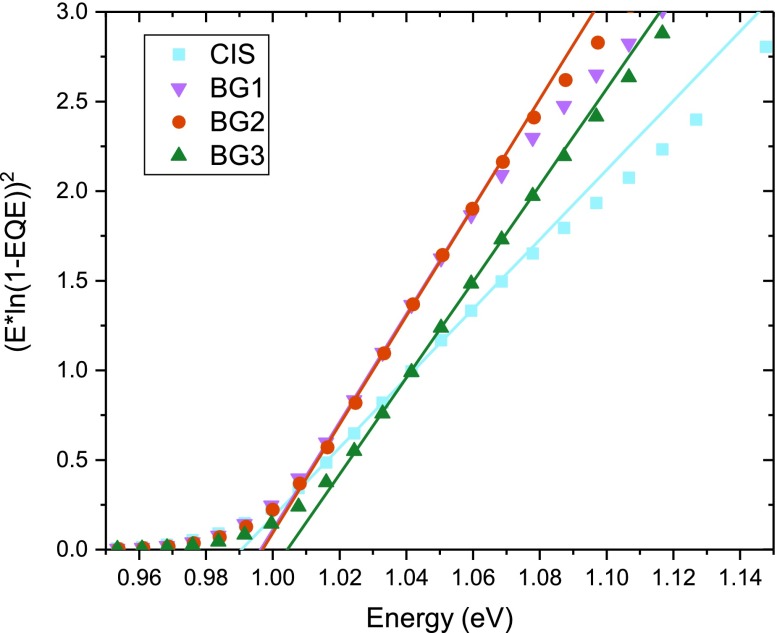
Bandgap extraction by Tauc fit to the external quantum efficiency. All absorbers show a bandgap around 1.00 eV.

A Tauc fit of the EQE results in bandgaps of 0.99 eV for the pure CIS, 1.00 eV for BG1 and BG2 and 1.01 eV for BG3 (Figure 5). The different slope between the graded cells and the CIS sample can be attributed to the differences in collection already seen in the EQE measurements. As a result the extraction of bandgap from EQE data may be questionable, especially for the non-graded case, but very similar results were extracted from optical transmission measurements on the absorber, showing a bandgap difference of about 0.02 eV between CIS and the back graded samples (see SI F2).

**Figure 6.**
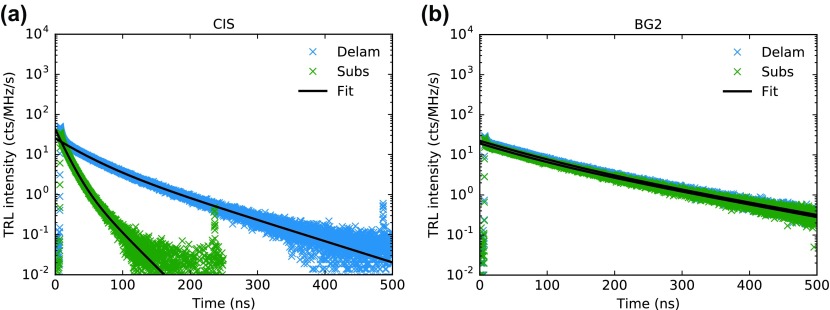
TRPL transients of absorbers without (a) and with (b) back grading. The samples were measured as grown (Subs) and after delamination from the back contact (Delam).

In order to compare the influence of back surface recombination and other competing processes, TRPL was measured for the pure CIS and BG2 (Figure 6). Both absorbers were measured as grown (on molybdenum) and after delamination of the back contact.

The ungraded CIS displays considerably higher apparent lifetimes when delaminated from the Mo back contact, indicating a reduction in back surface recombination. For the back graded sample on the other hand the same procedure has almost no influence on decay times and both samples show slower decays than even the lifted sample in the CIS case. These results indicate that the improvements seen are a combination of reduced back surface recombination and improved bulk absorber quality. A more detailed discussion of the TRPL behavior of these samples together with a model to estimate for the relative recombination rates at the back contact compared to the bulk recombination are shown elsewhere [20].

Temperature dependent *J*_SC_-*V*_OC_ measurements were performed at different illumination intensities to investigate recombination characteristics [21].

**Figure 7.**
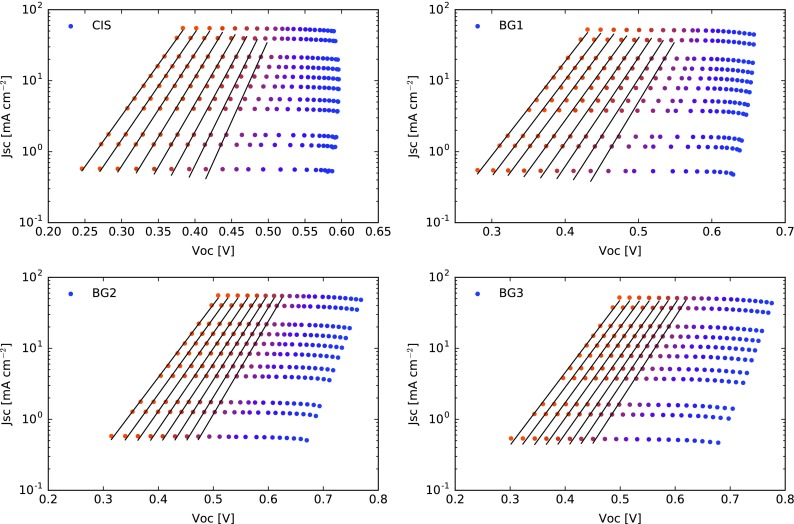
*J*_SC_-*V*_OC_ measurements at different temperatures and light intensities. The investigated temperatures range from 123 K (blue) to 323 K (orange). The lines show the linear fit for temperatures above 250 K.

The curves in Figure 7 show that the samples with no or small back grading (CIS, BG1) display a reduced *V*_OC_ for illumination close to 1 sun or above at low temperatures. This kind of behavior has been discussed in literature as being a result of a non-ohmic back contact or high back contact recombination [22]. Additional artifacts can be caused by heating of the sample during the measurement due to the increasing light intensity. In order to reduce the influence of these *V*_OC_ reductions on the analysis we only take the data points above 250 K into account for further parameter extraction.

**Figure 8.**
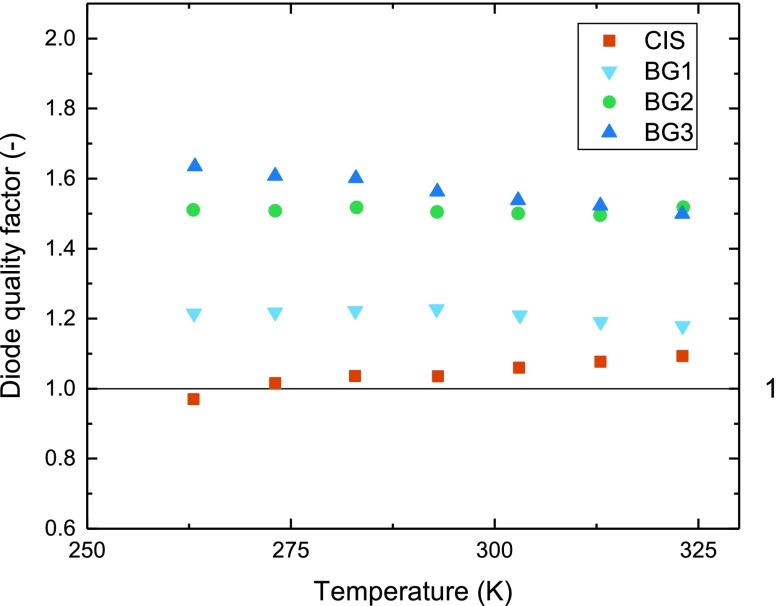
Diode quality factor dependence on temperature, extracted from *J*_SC_ (*V*_OC_) measurements.

As shown in Figure 8, the pure CIS cell as well as the cell with a flat grading (BG1) show diode quality factors (A) close to 1 while for the cells with stronger grading it increases towards 1.5. In the measured region, the diode quality factor is mostly independent of the temperature. We have extracted the activation energy for the dominant recombination path from *V*_OC_-*T* measurements and summarized the data in Table 3.

**Table 3.** Electronic parameters extracted from *V*_OC_-*J*_SC_ and *V*_OC_-*T* (SI F3) measurements. *A* is given at 293 K.

	*A* (–)	*E*_A, VocT_ (eV)
CIS	1.01	0.97
BG1	1.14	1.00
BG2	1.46	1.07
BG3	1.50	1.07

The increasing ideality factor with stronger back surface grading indicates a shift of the dominating recombination path from the quasi neutral region towards the space charge region [23]. The reduced activation energy for the cells without or only weak back grading could be attributed to the influence of back interface recombination. It should be noted that the activation energies for the strongly graded cells (BG2 + BG3) lie above the bandgap determined from the EQE. We assume that all EQE bandgaps are measured slightly lower because of the influence of tail states on the optical absorption [24].

**Figure 9.**
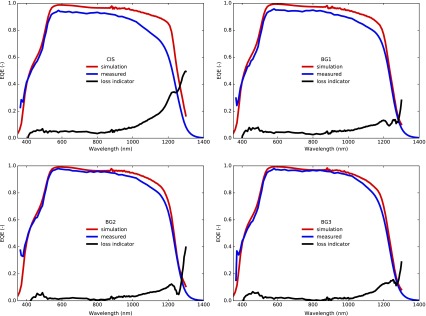
Measured (blue) and simulated (red) EQE for cells with and without back grading. The black lines represent (1 − EQE/simulation), an inverse measure for the collection efficiency.

In order to identify the remaining current losses in the back graded cells we compared the measured quantum efficiency with a simulated EQE based on optical measurements (Figure 9). To do so we modeled the light propagation in a multilayer solar cell by using the transfer matrix method formalism following the approach of Ref. [24], which notably takes into account reflectance and parasitic absorption in window layers. The compositional gradings of Figure 1 were discretized in 25 nm thick slices and the EQE was computed as the cumulated absorption in each of the CIGS slices, assuming perfect collection of the charge carriers. The validity of the CIGS absorption spectrum was assessed by comparing simulated and experimental absorption spectra for the corresponding delaminated absorbers. The difference between simulated and experimental EQE can be attributed to the charge carrier collection losses in the device.

The improvement in collection of carriers in the long wavelength region is clearly visible and already significant for the weakest grading (BG1). A non-negligible collection loss in the NIR remains even for the stronger gradings, but the EQE shape becomes primarily determined by optical absorption losses which do not affect the cells electronic properties. We also see a drop in EQE maximum for the cells with no or little grading, which could indicate a transport barrier in those cells. For further improvements, a reduction of parasitic absorption and reflection losses will become important.

To evaluate the suitability of the back-graded cells for their use in tandem devices with a perovskite top cell we calculated the expected bottom cell current density, for simplicity in a 4-terminal tandem device.

**Figure 10.**
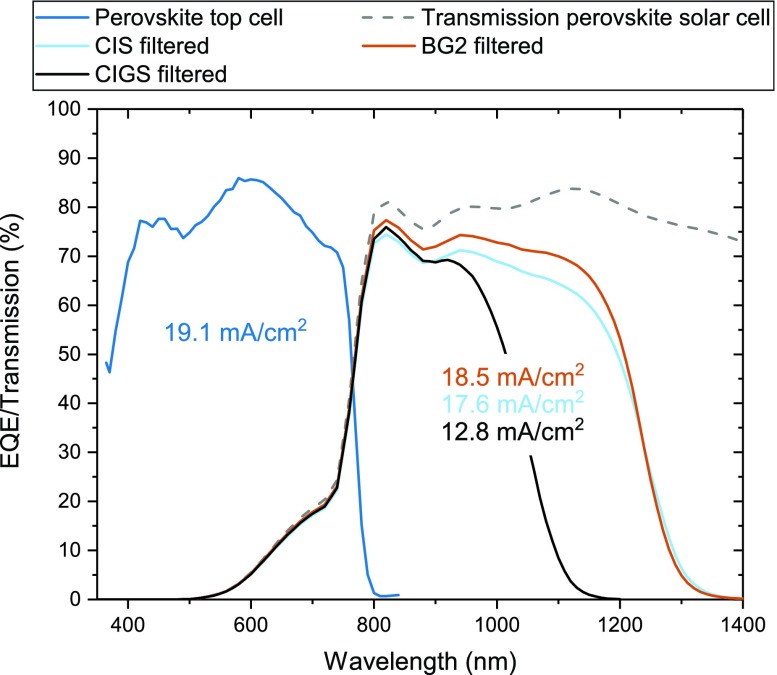
Calculated external quantum efficiency of BG2 in a 4-terminal tandem device. The improved collection seen in Figure 4 leads to a considerable improvement in bottom cell current density compared to the pure CIS. Perovskite EQE and transmission data are taken from [7].

Figure 10 shows that the CIS cells with back grading are expected to almost reach current matching to the perovskite top cell, a considerable improvement over the state of the art CIGS and notably higher than the reference pure CIS. With at least one absorbing transparent contact less, we expect such cells to get even closer to current matching in monolithic configuration.

## 4. Conclusions

We have shown that the efficiency of narrow bandgap CIS can be improved considerably by integrating a bandgap grading towards the back. The resulting cells show an improved collection of charge carriers in the NIR region while the EQE bandgap remains unchanged with an absorption onset at 1.0 eV, which is most relevant for bottom cells in current-matched 2-terminal tandem devices.

The combination of improved bulk and effectively reduced back surface recombination leads to a high *V*_OC_ of almost 560 mV for the cells with bandgap grading towards the back shown here. Highest efficiency of 16.1% has been demonstrated for a cell with optical bandgap of 1.0 eV. Such cells are the ideal partner for monolithic thin film tandem devices and will allow current matching with top-cells with wide bandgap absorbers (1.7 eV), as for example halide-based perovskite devices.

## Disclosure statement

No potential conflict of interest was reported by the authors.

## Funding

This work was financially supported by the Swiss National Science Foundation (SNF)-NRP70, PV2050 [project numbers 407040_153976; 407040_153916]; SNF-NanoTera and the Swiss Federal Office of Energy [SYNERGY: 20NA21_150950], as well as the Competence Centre for Energy and Mobility (CCEM CONNECT-PV).

## Supplemental data

Supplemental data for this article can be accessed at https://doi.org/10.1080/14686996.2018.1444317.

## Supplementary Material

Suppl.zip
