# Supplementary material for: Single-graded CIGS with narrow bandgap for tandem solar cells
Source: Sci Technol Adv Mater. 2018 Mar 16;19(1):263–70. doi: 10.1080/14686996.2018.1444317 (PMC5917437; doi:10.1080/14686996.2018.1444317)
Supplement: Suppl.zip [file TSTA_A_1444317_SM6714.zip › Feurer_Single-graded CIGS with narrow bandgap for tandem solar cells_revised_Suplementary_information.docx]

**Single-graded CIGS with narrow bandgap for tandem solar cells**

Thomas Feurer *, Benjamin Bissig, Thomas Paul Weiss, Romain Carron, Enrico Avancini, Johannes Löckinger, Stephan Buecheler and Ayodhya N. Tiwari

Laboratory for Thin Films and Photovoltaics, Empa - Swiss Federal Laboratories for Materials Science and Technology, Ueberlandstrasse 129, 8600 Duebendorf, Swit-zerland

*corresponding author: Thomas Feurer, thomas.feurer@empa.ch

Acknowledgements:

Financial funding from the Swiss National Science Foundation (SNF)-NRP70, PV2050 (project NO.: 407040_153976 and 407040_153916), SNF-NanoTera and the Swiss Federal Office of Energy (SYNERGY: 20NA21_150950), as well as the Competence Centre for Energy and Mobility (CCEM CONNECT-PV) are gratefully acknowledged.

ORCID:

Thomas Feurer 0000-0002-0281-0176

Benjamin Bissig 0000-0001-9066-027X

Thomas P. Weiss 0000-0003-1823-4481

Romain Carron 0000-0001-8281-4881

Enrico Avancini 0

Johannes Löckinger 0000-0002-8154-3511

Stephan Buecheler 0

Ayodhya N. Tiwari 0

Single graded CIGS with low band gap for tandem solar cells

# Supplementary information


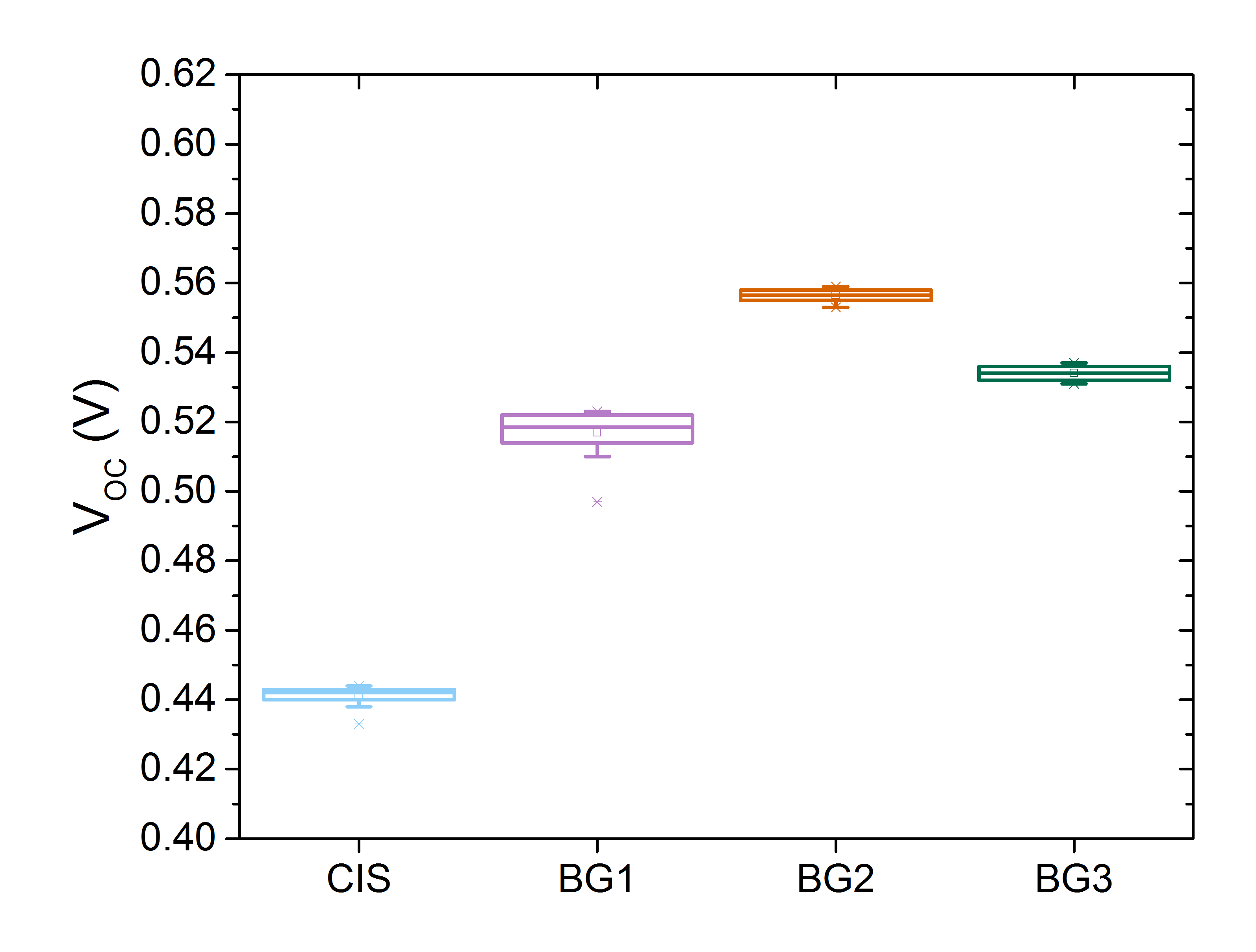

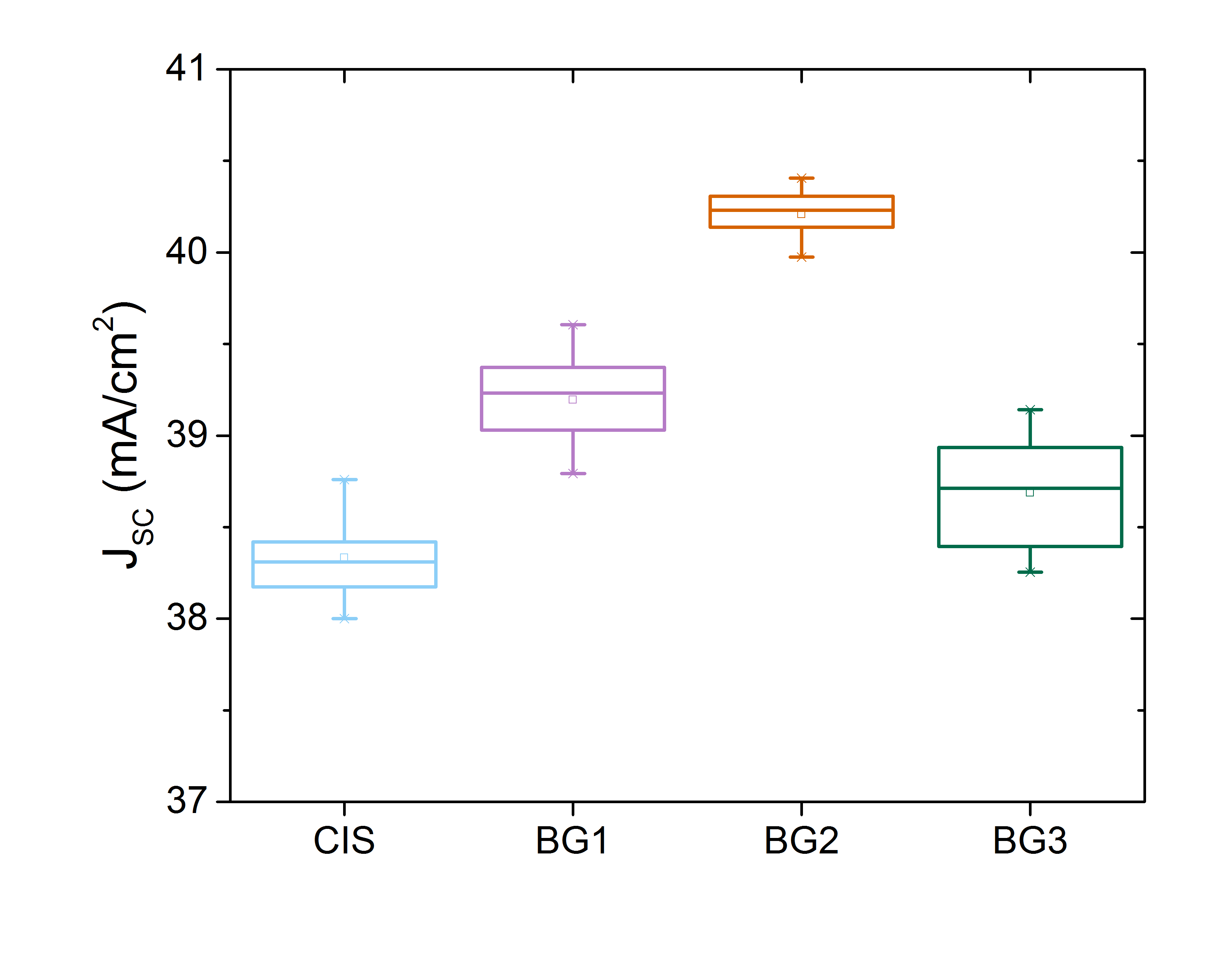

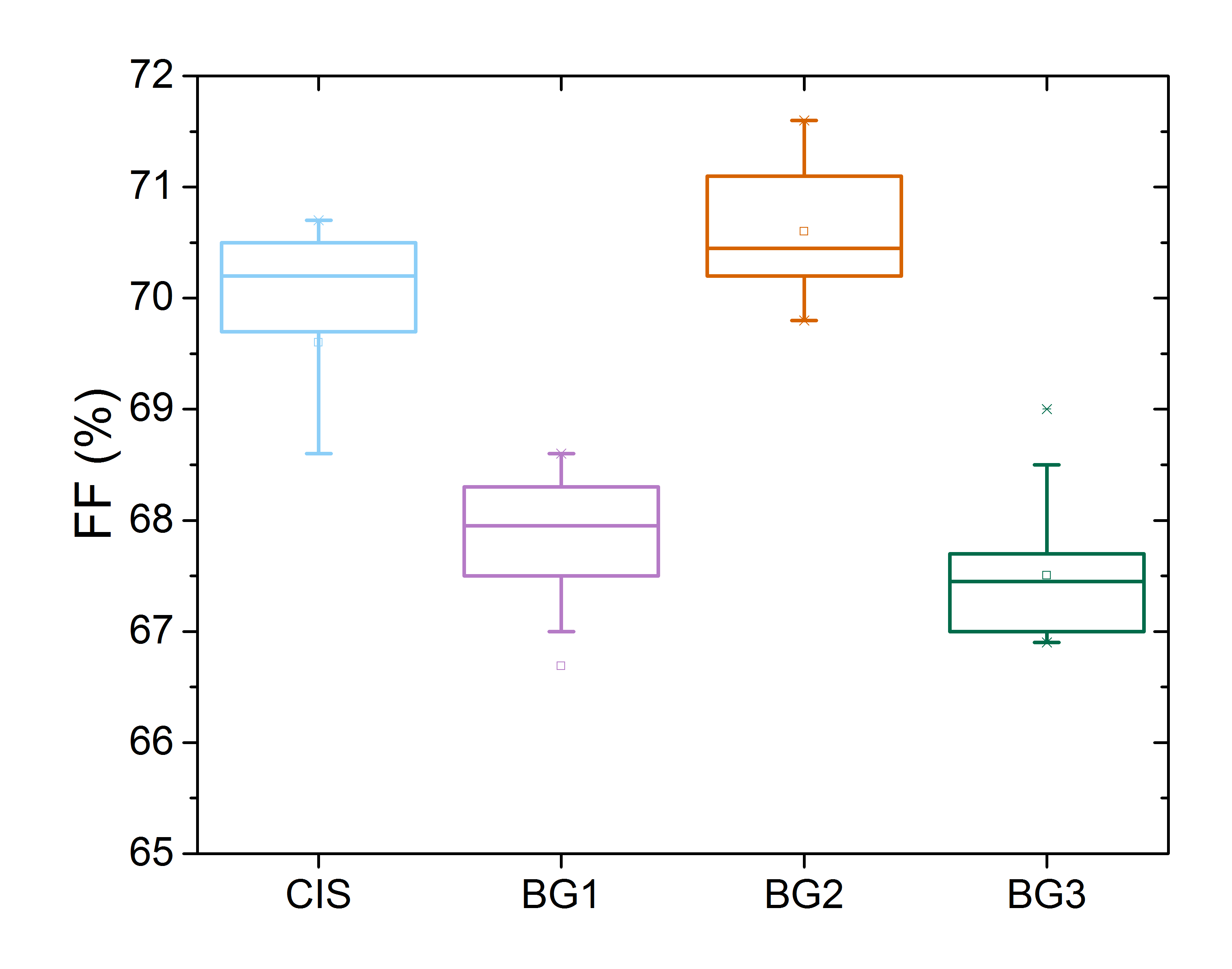

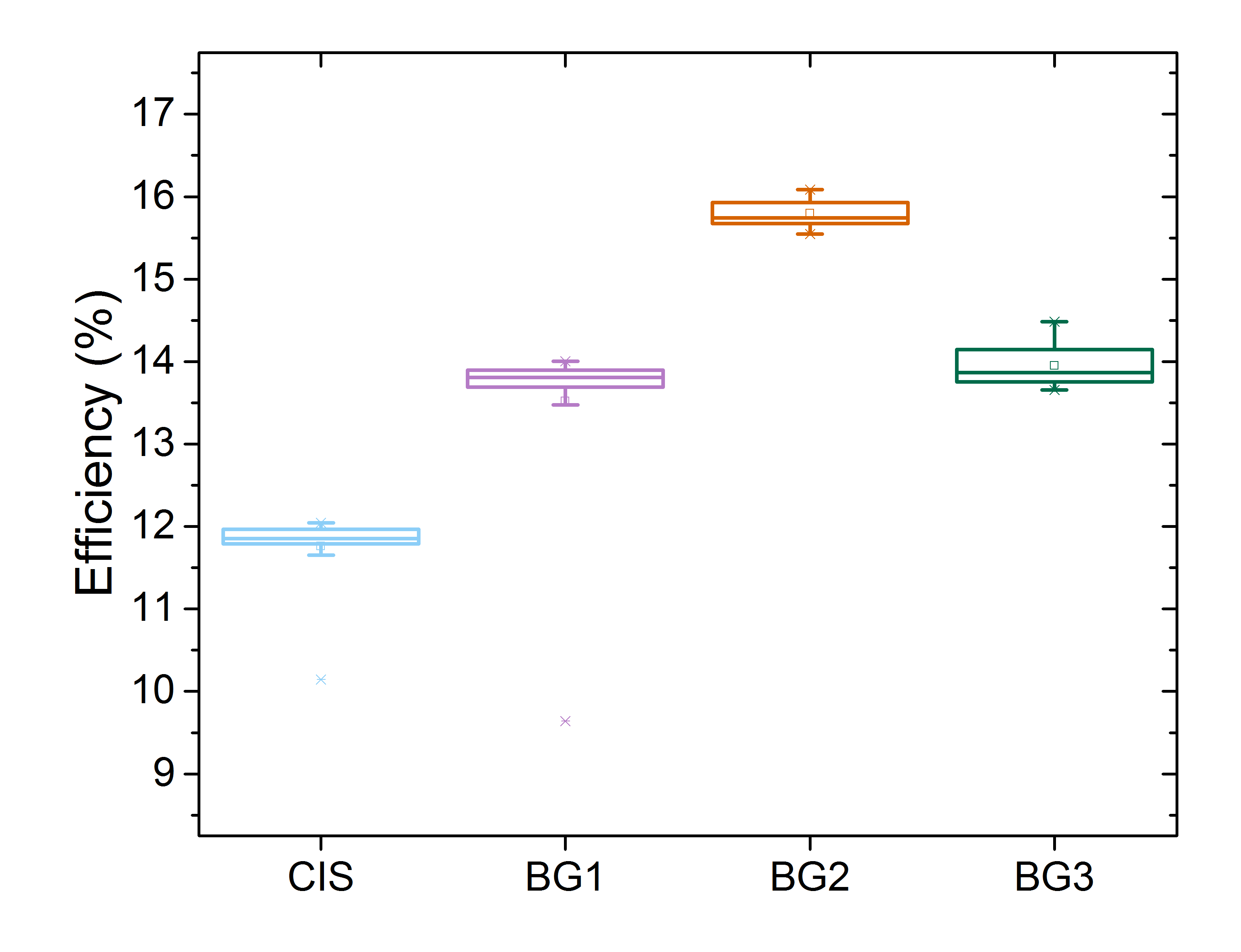


Supplementary Figure 1: JV- parameters of the samples with back grading in this work. Statistics over 18 cells each.

Supplementary Figure 2: Absorption of lifted of absorbers and representing bandgap extraction. The Absorption is extracted as 1-T/(1-R). The values do not reach 0 due to light-trapping inside the layer during the measurement.


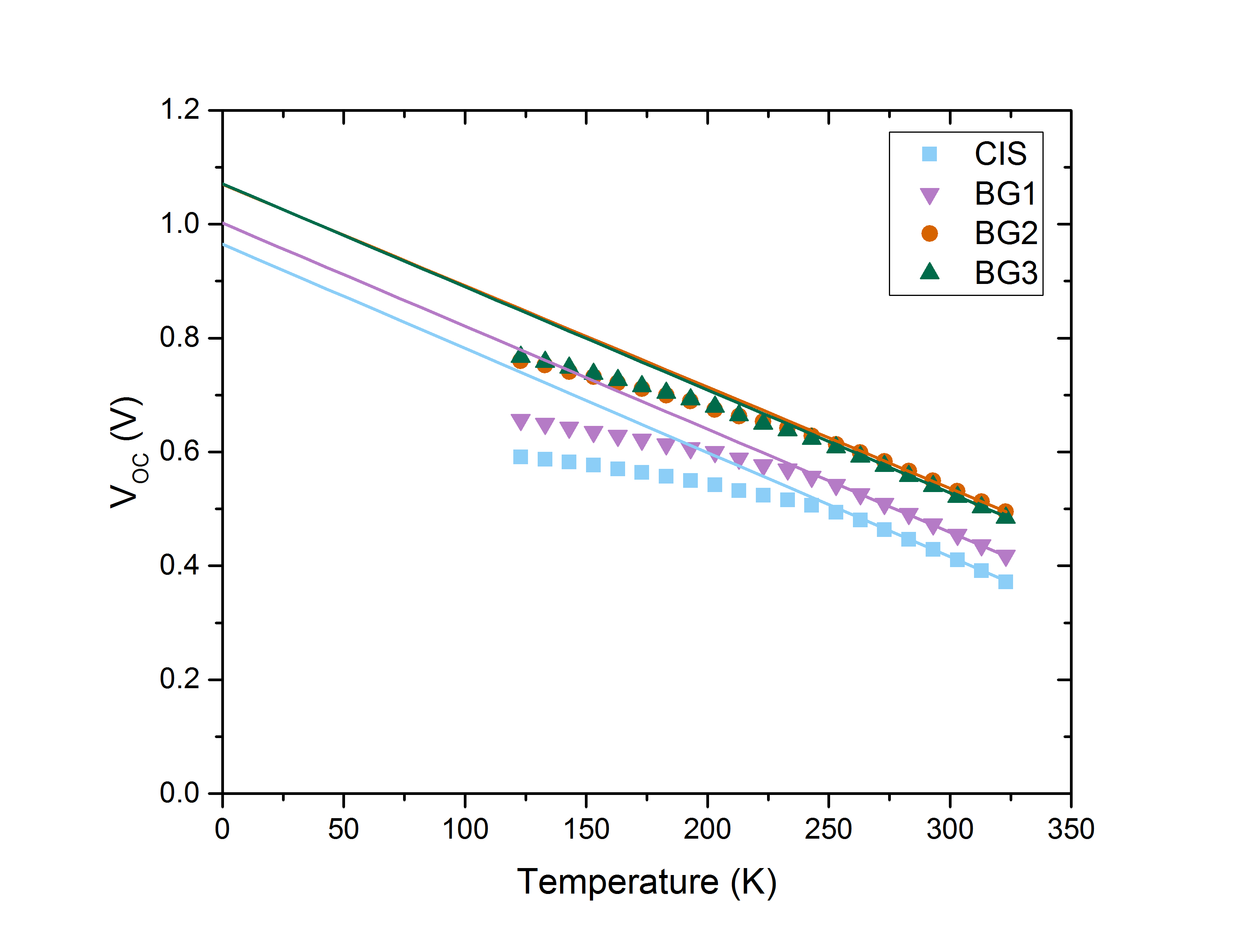


Supplementary Figure 3: V_OC_-T plot for the cells investigated in this experiment. The extrapolation of the linear region gives activation energies below bandgap for the non- or only weakly graded cells

**Tables**

*Supplementary Table 1:* Fitting results of the IV curves presented in Fig. 1 using a one-diode model including series and parallel resistances. Rs_illu_ and Rp_illu_ is fitted on the illuminated curve and averaged over 16 or more cells while Rp_dark_, J_0_ and A are fitted on the dark *JV* curve.

|  | GGI | Rs_illu_  (Ω cm^2^) | Rp_illu_  (Ω cm^2^) | Rp_dark_  (Ω cm^2^) | J_0_  (mAcm^-2^) | A  (-) |
| --- | --- | --- | --- | --- | --- | --- |
| CIS | 0.00 | 0.33 | 510 | 4600 | 2.05E-5 | 1.24 |
| BG1 | 0.04 | 0.23 | 460 | 1600 | 4.71E-5 | 1.54 |
| BG2 | 0.06 | 0.35 | 1240 | 2800 | 5.42E-5 | 1.66 |
| BG3 | 0.09 | 0.23 | 530 | 2500 | 1.19E-4 | 1.71 |
