# Supplementary material for: Single-graded CIGS with narrow bandgap for tandem solar cells
Source: Sci Technol Adv Mater. 2018 Mar 16;19(1):263–70. doi: 10.1080/14686996.2018.1444317 (PMC5917437; doi:10.1080/14686996.2018.1444317)
Supplement: Suppl.zip [file TSTA_A_1444317_SM6714.zip › Suppl.pdf]

Single-graded CIGS with narrow bandgap for tandem solar cells

Thomas Feurer *, Benjamin Bissig, Thomas Paul Weiss, Romain Carron, Enrico Avancini, Johannes Löckinger, Stephan Buecheler and Ayodhya N. Tiwari

Laboratory for Thin Films and Photovoltaics, Empa - Swiss Federal Laboratories for Materials Science and Technology, Ueberlandstrasse 129, 8600 Duebendorf, Switzerland

*corresponding author: Thomas Feurer, thomas.feurer@empa.ch

Acknowledgements:

Financial funding from the Swiss National Science Foundation (SNF)-NRP70, PV2050 (project NO.: 407040_153976 and 407040_153916), SNF-NanoTera and the Swiss Federal Office of Energy (SYNERGY: 20NA21_150950), as well as the Competence Centre for Energy and Mobility (CCEM CONNECT-PV) are gratefully acknowledged.

ORCID:

Thomas Feurer	0000-0002-0281-0176
Benjamin Bissig	0000-0001-9066-027X
Thomas P. Weiss	0000-0003-1823-4481
Romain Carron	0000-0001-8281-4881
Enrico Avancini	0
Johannes Löckinger	0000-0002-8154-3511
Stephan Buecheler	0
Ayodhya N. Tiwari	0

Single graded CIGS with low band gap for tandem solar cells

Supplementary information

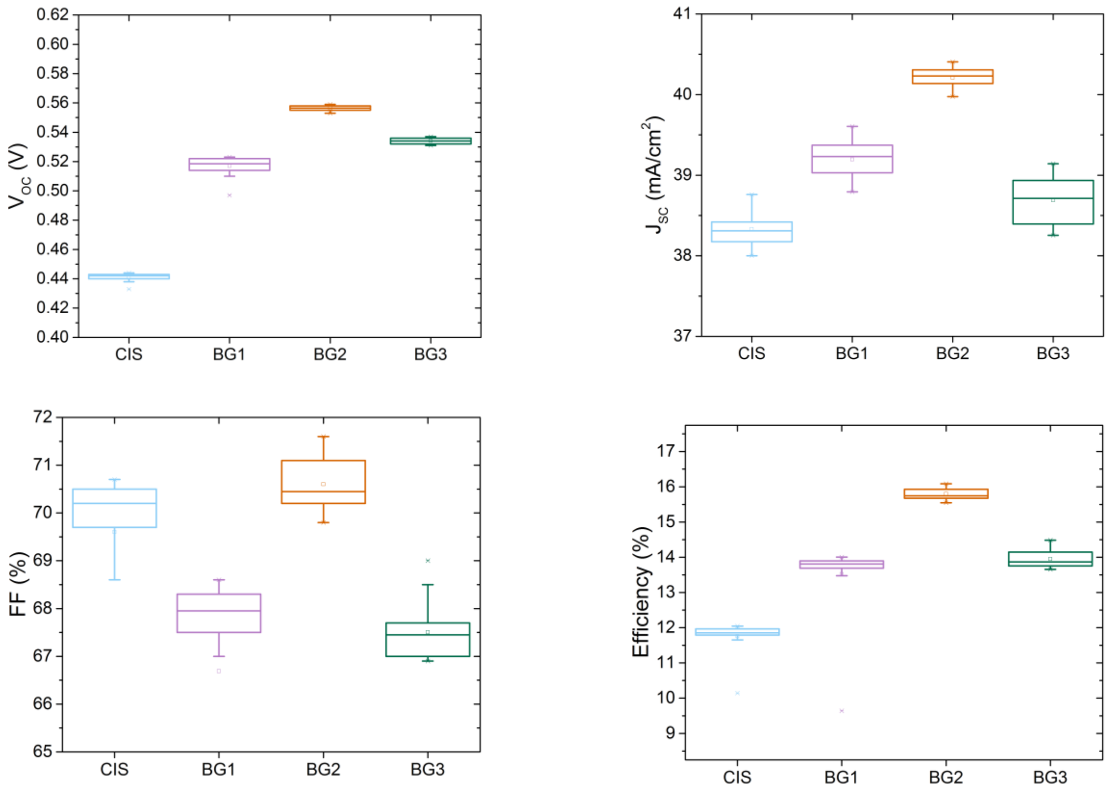

Supplementary Figure 1: JV- parameters of the samples with back grading in this work. Statistics over 18 cells each.

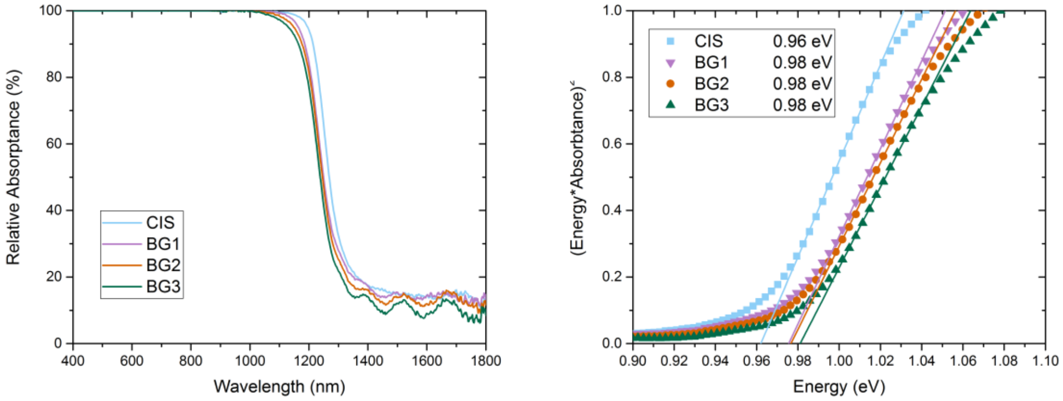

Supplementary Figure 2: Absorption of lifted of absorbers and representing bandgap extraction. The Absorption is extracted as 1-T/(1-R). The values do not reach 0 due to light-trapping inside the layer during the measurement.

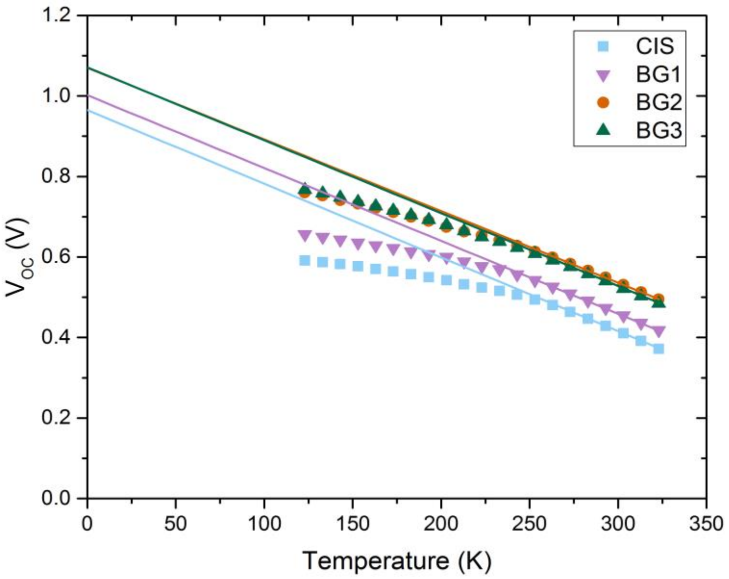

Supplementary Figure 3: V_{OC}-T plot for the cells investigated in this experiment. The extrapolation of the linear region gives activation energies below bandgap for the non- or only weakly graded cells

Tables

Supplementary Table 1: Fitting results of the IV curves presented in Fig. 1 using a one-diode model including series and parallel resistances. Rs_{illu} and Rp_{illu} is fitted on the illuminated curve and averaged over 16 or more cells while Rp_{dark}, J_0 and A are fitted on the dark *JV* curve.

	GGI	Rs_{illu} (Ω cm^2)	Rp_{illu} (Ω cm^2)	Rp_{dark} (Ω cm^2)	J_0 (mAcm^{-2})	A (-)
**CIS**	0.00	0.33	510	4600	2.05E-5	1.24
**BG1**	0.04	0.23	460	1600	4.71E-5	1.54
**BG2**	0.06	0.35	1240	2800	5.42E-5	1.66
**BG3**	0.09	0.23	530	2500	1.19E-4	1.71
